# Flaviviruses—Induced Neurological Sequelae

**DOI:** 10.3390/pathogens14010022

**Published:** 2024-12-31

**Authors:** Samantha Gabrielle Cody, Awadalkareem Adam, Andrei Siniavin, Sam S. Kang, Tian Wang

**Affiliations:** 1 Department of Microbiology & Immunology, University of Texas Medical Branch, Galveston, TX 77555, USA; sgcody@utmb.edu (S.G.C.); awadam@utmb.edu (A.A.); aesiniav@utmb.edu (A.S.); saskang@utmb.edu (S.S.K.); 2 Institute for Translational Sciences, University of Texas Medical Branch, Galveston, TX 77555, USA; 3 Sealy Institute for Vaccine Sciences, University of Texas Medical Branch, Galveston, TX 77555, USA; 4 Institute for Human Infections and Immunity, University of Texas Medical Branch, Galveston, TX 77555, USA; 5 Department of Pathology, University of Texas Medical Branch, Galveston, TX 77555, USA

**Keywords:** flavivirus, neurotropic, neurological sequelae, animal models

## Abstract

Flaviviruses, a group of single-stranded RNA viruses spread by mosquitoes or ticks, include several significant neurotropic viruses, such as West Nile virus (WNV), Japanese encephalitis virus (JEV), tick-borne encephalitis virus (TBEV), and Zika virus (ZIKV). These viruses can cause a range of neurological diseases during acute infection, from mild, flu-like symptoms to severe and fatal encephalitis. A total of 20–50% of patients who recovered from acute flavivirus infections experienced long-term cognitive issues. Here, we discuss these major neurotropic flaviviruses-induced clinical diseases in humans and the recent findings in animal models and provide insights into the underlying disease mechanisms.

## 1. Introduction

Flaviviruses are a group of single-stranded RNA viruses, many of which are transmitted by mosquitoes or ticks. Among them, there are several major neurotropic viruses, including Japanese encephalitis virus (JEV), Powassan virus (POWV), tick-borne encephalitis virus (TBEV), West Nile virus (WNV), and Zika virus (ZIKV). These viruses cause a range of neurological diseases in humans during the acute infection phase, from mild, flu-like symptoms to severe and fatal encephalitis [1,2,3,4]. Additionally, 20–50% of patients who recover from acute flavivirus infections experience long-term cognitive issues [4,5,6]. At present, only a few vaccines are approved for human use, and antiviral treatments are still being developed so treatment for flaviviral infections remains primarily supportive. Here, we discuss the neurotropic flaviviruses-induced clinical diseases in humans and recent findings in animal models, with a focus on the virus-induced neurological sequelae. 

## 2. Neurotropic Flaviviruses

### 2.1. JEV

#### 2.1.1. JEV Infection in Humans

JEV is a neurotropic flavivirus that is endemic in many parts of Asia and the Western Pacific. Humans are dead-end hosts infected by *Culex* mosquitoes, which circulate the virus between amplifying hosts such as pigs and wading birds [7]. Clinical manifestations of acute JEV infection in humans range from mild fever to severe encephalitis and mortality. 

Up to 50% of JEV patients experience long-term neurological complications [8,9]. Some convalescent JEV patients developed persistent fatigue two and a half years post-infection with areflexia, abnormal mouth movements, and dilated pupils. Diminished IQ, adaptive behavior impairment, motor neuron symptoms, epilepsy, and mental disorders were also documented [10,11]. A case study in Gansu, China, confirmed that JEV patients continued to display neurological abnormalities up to eight years after their initial hospital discharge [12]. A decade-long study in India based on several hundred laboratory-confirmed cases reported that 2 to 5% of patients presented with psychological disturbances, memory impairment, or disturbed speech, while other patients had significant improvement during the course of the study [13]. JEV-induced neurological sequelae occurred more frequently in children with a variety of clinical outcomes. For example, a study of 63 JEV-infected children in China reported that five males relapsed during the convalescent phase due to the induction of anti-N-methyl-D-aspartate receptor encephalitis [14]. In another case, a 10-year-old JEV patient in New South Wales, Australia, showed weakness in the right arm, hypophonic speech, mood instability, and impulsivity and required nasogastric feeding due to dysphagia following a 12-week hospitalization. At the 11-month follow-up, the patient remained to have mild disability due to dysphagia [15]. An 18-year-old JEV patient in Liaoning, China, who received JEV vaccination as a child, developed extensive lower limb weakness and Guillain–Barre Syndrome (GBS) after JEV infection. The patient later succumbed to JEV infection 36 days after symptom onset due to refusal of medical treatment [16]. An 11-year-old female from West Bengal, India, developed mesencephalon-thalamic encephalitis three weeks after acute JEV symptom onset. One year later, the patient experienced axial rigidity and occasional dystonic posturing of the upper limbs [17]. 

Among a group of 323 laboratory-confirmed cases of JEV, anti-JEV IgM antibodies were detected in the cerebrospinal fluid (CSF) of nine individuals 50 to 180 days after their first experienced symptoms. Viral antigen was also detected in the CSF up to 117 days after their initial illness, suggesting the persistence of viral infection [18]. In vitro JEV infection in human monocytes produced a persistent infectious virus for over three weeks [19]. Thus, persistent JEV infection long after the acute phase may play a role in the development of neurological sequelae. 

#### 2.1.2. Animal Models of JEV Infection

Studies in young C57BL/6 (B6) mice and macaques showed that the virus triggered neuronal apoptotic cell death and induction of the production of proinflammatory cytokines and chemokines in the brain, which contribute to the activation of astrocytes and microglial, the recruitment of inflammatory infiltrates, and blood–brain barrier (BBB) breakdown [20,21]. JEV-infected B6 mice presented with motor deficits 4 to 16 days post-infection (DPI) with symptoms including stiffness, tail dragging, limb paralysis, and missteps [22]. Intracranial infection of 12-day-old Wistar rats with JEV resulted in a significant decline in spatial memory at 10- and 33 DPI but improved at 48 DPI [23]. Treatment with Atorvastatin in JEV-infected BALB/c mice starting two hours post-infection for five times daily reduced viral loads, mitigated neuronal progenitor cell death in the subventricular zone of the brain, and limited inflammatory cytokine and chemokine production in microglial cells. Atorvastatin was proposed to reduce the levels of phosphorylated NF-κB in infected microglial cells and thus inhibited the production of proinflammatory cytokines [24]. JEV infection was reported to persist longer in the pregnant mice for 16 weeks compared to 4 weeks in non-pregnant mice, suggesting a role of pregnancy-associated immunosuppression in the delay of viral clearance [25]. Similar to its infection in human microglial cells, JEV infection in mouse microglial cell lines also produced a persistent infectious virus [26]. Acute JEV infections were also characterized in hamsters. Baby hamsters infected via the parenteral route developed viremia and high mortality [27]. While the majority of adult hamsters survived systemic epidemic JEV strain infection, they developed viremic and encephalitic lesions but failed to display overt clinical signs of disease, which is similar to what was observed in acute human diseases [28]. Follow-up studies are needed in investigating the outcome of long-term neurological sequelae in hamsters.

### 2.2. TBEV and POWV

#### 2.2.1. TBEV and POWV Infection in Humans

##### TBEV Infection in Humans

Tick-borne encephalitis (TBE) is a zoonotic disease caused by TBEV [29]. The virus was first isolated in 1937 and has been a leading cause of virus-induced neurological diseases in Europe and Asia [30]. TBEV is transmitted primarily via the bite of infected ticks, such as *Ixodes ricinus* and *Ixodes persulcatus*, but can also be transmitted through unpasteurized dairy products from infected livestock [31]. There are three main subtypes of TBEV: European, Siberian, and Far Eastern [32]. Two other subtypes have been recently identified, including the Baikalian and the Himalayan subtypes [33,34].

Approximately 5000 to 10,000 cases of TBEV infections are reported from endemic countries annually. Clinical symptoms of TBEV infection range from asymptomatic, mild, febrile headache, to a classic biphasic course, with a prodromal and subsequent neuroinvasive phases. About 50% of TBEV patients developed meningitis or encephalitis [35,36]. Rhombencephalitis and myeloradiculitis were also reported in TBEV patients. Rhombencephalitis is an inflammatory disease affecting the hindbrain and is associated with significant mortality [37,38]. Sporadic cases of gastrointestinal dysfunction were documented for some TBEV patients. The symptoms were often associated with the first viremic phase and resulted from severe neurological impairment of the CNS, though direct evidence of infection of the enteric nervous system was not confirmed [39,40]. Most patients who recovered from the disease required further treatment due to long-term neurocognitive impairment and neuropsychological sequelae associated with the postencephalitic syndrome [41]. While the mortality rate of TBEV infections is 1–4%, the Far Eastern subtype induces a more severe course with a mortality rate of up to 35% [30,42]. Chronic TBE has not been well studied and is mostly associated with infection by the Siberian and Far Eastern subtypes. There are no specific antiviral drugs against TBE. Vaccination remains a highly effective strategy in preventing TBEV infection.

TBEV-induced postencephalitic syndrome can cause long-term morbidity that is associated with various neurological and neuropsychiatric dysfunctions. The most common manifestations are cognitive disorders and neuropsychiatric complaints such as apathy, irritability, memory disorders, disturbances of vision, headache, hearing loss or tinnitus, coordination problems, and flaccid paresis or paralysis [43]. A clinical study involving a cohort of patients from six European countries between 2010 and 2017 with a confirmed diagnosis of 533 TBEV cases, among which 37.3% and 49.2% developed meningitis and meningoencephalitis, respectively. About 20 to 50% of convalescent TBEV patients displayed a wide range of neurocognitive and motor dysfunctions months to years after the infection, including headache, fatigue, decreased concentration, ataxia, tremors and paresis of extremities, memory problems, and sleep disturbances, reduced initiative/motivation, balance disturbances, coordination problems, learning difficulties, and problems with fine motor skills [6,44,45,46]. A retrospective analysis of 1072 TBEV patients showed that patients with meningoencephalomyelitis were predisposed to neurologic sequelae. Laboratory tests also revealed significantly elevated CSF protein concentrations in patients with neurologic sequelae [47,48]. Other risk factors, including male gender and older age at the time of disease onset, are linked with TBEV-induced long-term morbidity [49,50].

The major hallmarks of acute TBEV infection in the human brain include neuronal tropism, neuronal death, and astrogliosis. It was reported that in vitro TBEV infection in human neuronal/glial cells differentiated from neural progenitor cells recapitulated these events. This study also revealed that cell type-specific innate immunity was associated with TBEV tropism in human brain cells [51]. Elevated concentrations of macrophage migration inhibitory factor (MIF) and interleukin (IL)-1β in CSF and increased levels of serum tumor necrosis factor (TNF)-α, IL-6, and matrix metalloproteinase-9 (MMP-9) were observed in the TBEV patients [52,53]. MMPs are produced by various cells of the central nervous system (CNS) and can induce BBB permeability compromise, leading to meningoencephalitis and a number of neurological consequences [54]. Flow cytometry analysis of lymphocyte phenotype in the CNS of TBE patients revealed higher levels of T cells expressing HLA-DR during the early course of the disease, while T cells expressing higher IL-2 receptors appeared at later of infection. In comparison, B cells and NK cells were more profound in the peripheral blood. Furthermore, immunohistochemical analysis of brain tissue of TBEV patients showed granzyme B-expressing CD8^+^ T cells were in close contact with TBE-expressing neurons expressing high levels of caspase-3 in the brain parenchyma, suggesting a role of granzyme B-expressing CD8^+^ T cells in the induction of brain injury [55,56].

##### POWV Infection in Humans

POWV is another tick-borne neurotropic flavivirus. The virus was first isolated in 1958 from the brain of a child who died of encephalitis of unknown origin in the town of Powassan in Ontario, Canada [57]. Human cases of POWV infection were mostly reported in North America and Russia. The number of human cases of POWV encephalitis has increased in the past two decades, mostly in the United States [58]. Similar to TBEV infection, the most common clinical manifestations of POWV infection are encephalitis, meningoencephalitis, and aseptic meningitis [59,60]. Other clinical signs and symptoms of POWV infection include drowsiness, headache, disorientation, gastrointestinal involvement, focal neurologic signs, seizures, ataxia, twitching, tremors, lethargy, and paralysis [61,62]. The mortality rate of acute POWV infection is about 10%. Long-term neurological sequelae have been reported in at least 50% of surviving patients, which include cognitive deficits, speech difficulty, weakness, imbalance, hemiplegia, recurrent severe headaches, wasting, and memory problems [63,64]. Due to climate and socioeconomic changes, there have been increasing cases of POWV diseases reported in North America in recent years [65,66]. At present, no approved vaccines are available to control POWV infection in humans.

POWV is transmitted by *Ixodes scapularis* ticks, which are also the primary vector for the transmission of *Borrelia (B). burgdorferi*, the causative agent ofLyme disease [60,67]. POWV patients were often reported to have co-infection with other tick-borne pathogens, such as *B*. *burgdorferi*. Analysis of 106 serum/plasma samples collected from patients in Wisconsin identified 11 POWV seropositive samples, of which nine were from patients positive for Lyme disease [68]. A study conducted in New York and Connecticut found that the incidence of *B. burgdorferi*-POWV co-infection was as high as 2% [69]. The exact impact of co-infection *B. burgdorferi* in POWV-induced clinical diseases is still under investigation. A recent study by Hart et al. found that infection with *B. burgdorferi* enhanced the replication and dissemination of co-infecting POWV in the same ticks [70].

#### 2.2.2. Animal Models of TBEV and POWV Infection

TBEV: Laboratory animals play a crucial role in understanding the pathogenesis, immune response, and neurological consequences of TBEV infection. Systemic TBEV infection in immunocompetent mice, including B6, BALB/c, or C3H mice, led to the development of viremia, followed by weight loss, paralysis, and lethargy. Viral RNA was detected in various organs of the animals, with the highest amounts of RNA found in the brain, heart, and lungs. TBEV antigens were also detected in the gastric myenteric plexus and celiac plexus, indicating that the virus entered via the gastrointestinal autonomic nerves [71,72,73]. Studies in mice infected with the chimeric viruses incorporating components of TBEV and Omsk hemorrhagic fever virus (OHFV), another highly pathogenic tick-borne flavivirus that causes hemorrhagic fever, identified TBEV NS5 as a key player in promoting neuronal dysfunction and degeneration induced by TBEV infection [74]. Another study reported that TBEV hijacked the neuronal granule system to transport its viral RNA to dendrites, disrupting dendritic mRNA transport and causing impairment of neuronal functions such as neurogenesis and synaptic plasticity. Mutations in the 5′ untranslated region of TBEV genomic RNA inhibited the transport of the TBEV genome to dendrites and thus attenuated the neurological symptoms of TBEV in mice. Thus, the 5′ untranslated region of TBEV is associated with the transport of TBEV viral RNA in mouse neuronal dendrites and the development of neurological diseases [75]. TBEV infection in B6 mice also caused acute distension, segmental dilation of the intestinal tract, and enteric ganglioneuritis. The number of infiltrating immune cells was associated with the severity of ganglioneuritis, indicating immune-mediated damage to the enteric nervous system [76]. Induction of innate immune responses in the CNS plays a crucial role in the development and control of neurological diseases. For example, IPS-1 (IFN-β promoter stimulator I) promotes the regulation of expression of genes such as viperin and IRF-1 in wild-type mice, while the absence of IPS-1 results in higher viral replication, microglia/astrocyte activation, and increased proinflammatory response [77]. Intracerebral infection of BALB/c mice with TBEV resulted in an increase in RANTES levels and further destruction of brain tissue induced by brain infiltrates, suggesting a role of RANTES in mediating neuroinflammatory reactions and destruction of brain tissue [78]. Increased BBB permeability was observed at the late stages of TBEV infection and associated with a dramatic increase in the expression of a number of different cytokines and chemokines (TNF-α, IL-6, IFN-γ, RANTES, MCP-1, IP-10) in the brains of infected animals [79]. Overall, animal model studies suggest that CNS pathology during acute TBEV infection is a consequence of the activation of various types of immune cells, viral infection, and neuroinflammatory reactions. Proinflammatory cytokine-mediated increases in BBB permeability serve as one of the main mechanisms of virus entry into the CNS, leading to disease progression and potential long-term neurological consequences. Infected CNS cells are the source of proinflammatory cytokines in the brain and contribute to neurotoxicity and BBB disruption. 

POWV: Mouse models of POWV infection have been developed to study virus-induced CNS pathology and long-term neurological sequelae. Acute POWV infection in mice resulted in a range of neurological dysfunctions, including meningoencephalitis, poliomyelitis-like syndrome, loss of balance and mobility, hind limb paralysis, moribund state, and death. In addition, perivascular infiltration, apoptosis of Purkinje cells in the granular layer of the cerebellum, and the presence of viral antigen in the neurons of both the brain and spinal cord were reported. In the spinal cord, large motor neurons in the ventral horns were predominantly affected. In the cerebellum, Purkinje cells were the most highly infected by the virus [3]. Mice surviving POWV infection were often followed by the development of diverse chronic neurological dysfunctions ranging from weak grip strength, tremors, and seizures to flaccid paralysis. A weak grip, altered tail grasp reflex, and walking/positioning abnormalities were also observed, which recapitulated clinical symptoms such as areflexia and muscle weakness reported in POWV patients [3,80,81]. In an animal model of persistent POWV diseases, most infected B6 mice survived acute POWV infection with viral RNA persisting in the brain. Long-term neurological damage associated with POWV disease was observed in all surviving mice. The mice demonstrated meningitis, encephalitis, myelitis, gliosis, and neuronal necrosis, as well as infiltration of the meninges by various inflammatory cells. These data suggest that chronic inflammation in the CNS and persistence of viral RNA contribute to the virus-induced long-term neurological sequelae [82]. Furthermore, POWV-induced lethality consistently decreased with age. In the CNS of adult mice, POWV induced T helper (h)1-type cytokines (IFN-γ, IL-2, IL-12, TNFα, IL-6), indicating a neurodegenerative proinflammatory phenotype of M1 microglia. In contrast, in the more susceptible younger mice, POWV-induced Th2-type cytokines (IL-10, TGFβ, IL-4) corresponded to a neuroprotective phenotype of M2 microglia. Regardless of the age of the animals, infection in survivors resulted in long-term neurologic sequelae, including paralysis, ataxia, weak grip, and failure to right. Surviving mice showed reactive Iba1^+^ glial cells and persistent long-term CNS spongiform pathology, high levels of infiltrating CD4^+^ and CD8^+^ T cells in the CNS, microgliosis, and neuronal necrosis [83]. 

### 2.3. WNV

#### 2.3.1. WNV Biology and Human Infections

WNV was originally isolated in the West Nile Province of Uganda, Africa, in 1937. It was later spread to other parts of the world and became endemic in Africa, North and Central America, West Asia, and the Middle East [84]. WNV is transmitted by infected mosquitoes, primarily the *Culex* species [85]. There are nine defined WNV lineages, among which lineages 1 and 2 have been associated with the majority of reported human cases [86,87,88,89]. Lineage 1 has been detected in all continents except Antarctica. Lineage 2 is primarily found in Africa and Europe [86,87,88,89,90]. WNV continues to cause a threat to public health with yearly outbreaks. Despite multiple efforts contributing to developing WNV treatment and vaccines, no effective vaccines or antivirals to prevent or treat WNV infection in humans are currently available.

About 80% of WNV infections in humans are asymptomatic. Symptomatic human infections are characterized by high fever, headache, back pain, and neuroinvasive disease. WNV neuroinvasive disease includes meningitis, encephalitis, and acute flaccid paralysis [90,91]. A total of 20 to 60% of convalescent patients developed physical, cognitive, and functional sequelae, including depression, memory loss, motor dysfunction, severe fatigue, confusion, muscle weakness, and tremors, which lasted months to years after initial acute infection. Other behavior changes, including impaired balance, cerebellar ataxia, visual impairment, emotional instability, aggression, altered mental status, reduced activity levels, and employment alterations, were also reported in clinical case studies. New neurological abnormalities develop over time, regardless of the initial clinical presentation [92,93,94,95,96,97,98]. Certain groups were more vulnerable to WNV-induced severe neurological diseases or death. Specifically, male patients with preexisting conditions such as cardiovascular disease, cancer, kidney disease, diabetes, and hypertension often had a higher risk for acute WN neuroinvasive disease. WNV-induced sequelae were observed in individuals of all ages. The development of WNV neuroinvasive disease does not always correlate with the development of neurological sequelae. However, several factors, such as hypertension, diabetes, and age, have been associated with a high risk of induction of neurological sequelae [99,100].

#### 2.3.2. Animal Models of WNV Infections

Studies in hamsters and mice showed that WNV infects similar regions of the brain, spinal cord, and peripheral nerves as reported in humans. Thus, both models have been used to investigate the pathogenesis of WNV-induced neurological diseases [101,102]. WNV infection in mice, hamsters, and non-human primate (NHP) models all demonstrated the persistence of infectious virus and/or viral RNAs in the CNS tissues months following the initial WNV infection [102,103,104]. WNV persistence in the CNS was found to be associated with the virus-induced long-term neurological deficits in memory and motor function in hamsters [105,106]. In a murine model of WNV-induced cognitive dysfunction, Garber et al. reported that IL-1β produced by astrocytes impaired adult neurogenesis and thus promoted virus-induced memory loss [107]. Another study showed that WNV-induced memory loss was caused by microglial phagocytosis of presynaptic termini in the CA3 region of the hippocampus [5]. Furthermore, WNV-specific B cells and T cells were detected in the brains of persistently infected mice up to 16 weeks post-acute infection [108]. T cells, particularly CD8^+^ T cell-derived interferon-γ (IFN-γ) signaling induced activation in microglia, could trigger microglia-mediated synaptic elimination and cognitive dysfunction [109]. 

### 2.4. ZIKV

ZIKV is primarily transmitted by *Aedes* mosquitoes. ZIKV infection in humans commonly leads to a self-limiting febrile illness characterized by rash, joint and muscle pain, and conjunctivitis [110]. An outbreak in French Polynesia in 2013 was the first to report neurological sequelae following ZIKV infection in adults [111,112,113]. In November 2015, the incidence of neonatal microcephaly increased sharply in conjunction with the spread of ZIKV through Brazil and Latin America, leading to the identification of a novel pathology termed Congenital Zika Syndrome (CZS) [114,115,116]. In addition to CZS, ZIKV infection in adult patients resulted in mild to severe neurologic complications involving both central and peripheral nervous system disorders, such as GBS and other neurological sequelae [117,118]. At present, ZIKV continues to circulate in subtropical and tropical climates, and the expansion of the *Aedes* mosquito habitats may expose currently naïve populations to ZIKV in the upcoming years [119]. 

#### 2.4.1. ZIKV-Induced Neurological Sequelae in Adults

GBS is one of the most frequent peripheral nervous syndromes, which typically develops 1 to 2 weeks after acute ZIKV infection. GBS is the main cause of acute flaccid paralysis characterized by limb weakness, hypo- or areflexia, and proximally progressing paresthesia [120]. Clinical subtypes of GBS include acute peripheral neuropathy (APN), acute inflammatory demyelinating polyneuropathy (AIDP), acute motor axonal neuropathy (AMAN), acute motor sensory axonal neuropathy (AMSAN), and Miller Fisher Syndrome (MFS). Hospitalization, respiratory support, and long-term disability are all common outcomes of GBS [121]. Since its first association with ZIKV infection in French Polynesia, clinical evaluations of heterogeneous GBS subtypes have been documented in ZIKV patients, including AMAN, ASMAN, AIDP, and heterogenous subtypes. Scattered cases of MFS-subtype GBS were also documented in ZIKV patients [118,122,123,124,125,126,127,128,129,130,131,132,133,134,135,136,137,138,139,140]. Although GBS is generally considered a postinfectious pathology, some GBS symptoms occurred within a week of ZIKV symptom onset, suggesting a potentially parainfectious mechanism [128,134]. Follow-up studies of ZIKV-positive GBS cases reported chronic pain in 51% of patients at three months [118], neurological impairment in 40% of cases at one year post-discharge [125], and in 10% of cases two years post-hospital discharge [141]. Higher rates of disability were noted in ZIKV-positive GBS patients than in their counterparts with GBS from other infectious sources [142]. Differences in olfactory function were also noted, with ZIKV-positive GBS patients demonstrating significantly higher rates of hyposmia, lower olfactory thresholds, and a greater olfactory perception deficit than other GBS patients over a year after acute infection [143].

Other neurological manifestations have also been associated with ZIKV infection, including meningitis, encephalitis, meningoencephalitis, myelitis, sensory neuropathy, and Alice in Wonderland Syndrome [117,118,140,144,145,146]. Persistent neuropsychological and cognitive deficits were reported in an adolescent ZIKV patient, which was not fully resolved even 15 weeks after symptom onset [147].

#### 2.4.2. Animal Models of Adult Neurological Sequelae

Systemic ZIKV infection in aged interferon αβ-receptor knockout (IFNAR^−/−^) mice triggered temporary flaccid paralysis with motor neuron synaptic retraction, which recapitulated a GBS-like proximal peripheral neuropathy [148]. Treatment with memantine, an N-methyl-D-aspartate receptor antagonist, reduced the incidence of paralysis in the IFNAR^−/−^ mouse model [149]. Moreover, ZIKV-infected BALB/c mice showed motor impairment, ocular inflammation, demyelination in the hippocampus and motor cortex regions of the brain, and retinal/corneal hyperplasia even at 120 DPI. The observed pathology coincided with autoreactive antibodies targeting GD1a and GD1b gangliosides corresponding to human AMAN and sensory ataxic GBS presentations, respectively [150]. Garber et al. reported a murine model of ZIKV cognitive recovery in which adult immunocompetent B6 mice were inoculated intracranially with ZIKV. Infected animals displayed significant spatial learning deficits from 45 to 50 DPI. They found that IFN-γ-producing CD8^+^ T cells promoted neuronal apoptosis and microglial engulfment of postsynaptic termini and neurons in animals post-recovery of ZIKV infection [109]. In a similar adult mouse model, cognitive dysfunction was detected at 14 and 30 DPI but had returned to normal at 60 DPI. Activation of TNF-α, microgliosis, and upregulation of complement system proteins C1q and C3 were shown to be associated with ZIKV-induced synapse and memory dysfunction [151]. Furthermore, in rhesus macaques infected subcutaneously with ZIKV, systemic and CSF immune activation were associated with neurocognitive impairment detected at 14 and 28 DPI [152]. 

#### 2.4.3. Postnatal Neurological Sequelae

CZS is a pathology characterized by microcephaly, hypertonia, brain calcifications, and physical and cognitive deficits, which is present in 5–14% of the children exposed to ZIKV in utero [153,154]. Neurocognitive assessments have been performed in several cohorts of children born with CZS. Studies conducted between 1 and 2.5 years of age reported lower than average cognitive function and significantly lower mean age equivalents (MAE) in language, gross motor skills, fine motor skills, gross motor, receptive language, expressive language, and psychosocial development. In addition, poorer cognitive scores were found to be significantly correlated with smaller head circumference at birth [155,156,157]. Researchers evaluating cohorts of children with a mean age of 33.1 months showed severe cognitive delay in 55.5% and moderate cognitive delay in 16.7% of children within their cohort [158]. Due to the COVID-19 pandemic, several follow-up longitudinal studies in children born with CZS at the age of three were either lost or delayed. However, one recent report revealed that 85% of the 7 to 8-year-old cohort were severely impaired in their ability to communicate, while 68.3% of children were unable to effectively use their vision [159]. 

While the majority of children exposed to ZIKV in utero do not develop the CZS phenotypes, these termed “normocephalic” children demonstrate a unique neurodevelopmental profile compared to their microcephalic counterparts. In the assessment of normocephalic children, 15% presented with a developmental delay either in language or motor prior to their first birthday [160]. Two independent studies of children 12 to 24 months old noted delays in mobility, communication, and social cognition becoming more pronounced as the children aged [160,161]. A delay was also corroborated in a study of children spanning 7 to 32 months of age, where 28.1% of participants were below average cognitively and 12.3% were severely impaired in at least one aspect of neurodevelopment [162], though not all studies reported significance in delay at two years [163]. For children 24 to 36 months of age, researchers documented resolution of delay over time [164] and insignificant impairment compared to unexposed peers [165,166]. Though some studies reported low rates of mild delay around three years old [167,168,169], others reported no differences [170]. Studies reported no significant neurodevelopmental delays but noted increased rates of behavioral problems and difficulty with emotional regulation for children about five years old [171,172,173]. It was expected that the rates of autism diagnosis would be higher in children exposed to ZIKV in utero than in unexposed peers as well [173]. 

#### 2.4.4. Animal Models of Postnatal Neurological Sequelae

Several animal models have been developed to recapitulate ZIKV-induced postnatal neurological sequelae in humans. Following intravenous ZIKV infection in B6 dams at embryonic (E) 12.5, the adult offspring presented with normal sociability and object memory but abnormal social memory with increased grooming habits [174]. Amniotic fluid ZIKV infection of B6 dams at E15 produced pups with motor incoordination and visual dysfunctions associated with anatomical defects in the retina and cerebellar cortex at P (postnatal day) 40 [175]. Another study showed that male offspring born to low-dose ZIKV-infected mothers displayed more severe neuropathological alterations in the hippocampus and were more susceptible to learning and memory impairments compared to female offspring. Males also presented with an increased number of immature neurons in apical and basal hippocampal dendrites, while female offspring had immature neurons in basal dendrites [176]. In another study, 4 to 5-month-old female offspring of FVB/NJ dams intravaginally infected with ZIKV at E4.5 demonstrated a risk-taking behavior and hippocampal-dependent spatial memory deficit, which was mirrored by a significant reduction in syntaxin-1A and PSD95 expression in the hippocampus of female offspring but not in the males [177]. Immunocompromised mice were also used to model CZS. Four-week-old offspring born to IFNAR^−/−^ dams infected at E12.5 with ZIKV PRVABC59 strain displayed abnormalities in neuropsychiatric state, motor behavior, autonomic function, or reflex and sensory function [178]. 

Long-term neurobehavioral consequences have also been investigated in neonatal ZIKV infection animal models. In two studies [179,180], mouse strain and sex-dependent differences were identified in early susceptibility to the virus and subsequent CNS glial reaction as well as the long-term behavioral abnormalities developed later in adult mice, including hyperactivity, impulsiveness, and motor incoordination. In addition to its impact on social behavior abnormalities, neonatal exposure to ZIKV also resulted in infection in the hypothalamus, causing multi-hormone deficiencies and delayed growth and development in mice [181]. One study also reported that ZIKV-infected mice displayed autistic-like behaviors during adulthood [182]. To investigate the mechanisms of ZIKV-induced neurological sequelae, one group showed that ZIKV infection during early CNS development triggered long-lasting degeneration of neurons and activation of astrocytes, leading to subsequent loss of neurons and defective myelination [183]. In another study, ZIKV antigen was detected in the CNS of convalescent mice for up to one year, and the resulting neuroinflammation could increase the risk of long-term neurodegenerative disorders [184]. The prolonged treatment with anti-TNF-α monoclonal antibody Infliximab for 40 days, starting on the day following its neonatal infection, was found to prevent ZIKV-induced cognitive and motor impairments later at 60 DPI, suggesting a role of TNF-α-mediated inflammation in induction of ZIKV-induced long-term behavioral deficits [185]. 

NHP models were reported to recapitulate vision [186,187,188,189], hearing [186], and motor deficits [190] in humans. It was revealed that persistent enlargement of lateral ventricles in smaller volumes altered functional connectivity between brain areas, and neuropathological changes were associated with the behavioral and memory deficits observed one year following neonatal infection in rhesus macaques [191]. 

## 3. Summary and Future Perspectives

In summary, neurotropic flaviviruses induce neurological sequelae with diverse clinical outcomes. Among them, cognitive impairment is commonly observed. GBS was also reported in both JEV and ZIKV-induced neurological complications. The duration of the virus-induced neurological sequelae ranges from weeks to years post-acute onset. Older age and male gender have been identified to be the likely risk factors for some viruses. Animal models, such as murine models, have been developed to recapitulate virus-induced clinical symptoms in humans and have provided important insights into the disease mechanism. Persistent virus infection in the CNS residential cells, such as microglial cells, has been shown to be associated with flavivirus-induced long-term neurological sequelae. Neuroinflammation mediated by CNS residential and infiltrating peripheral immune cells also contributed to flavivirus-induced neurological changes. For example, microglial phagocytosis with the help of the infiltrating CD8^+^ T lymphocytes and complement activation in the CNS contribute to WNV and ZIKV-induced chronic cognitive dysfunctions and memory loss post-recovery. However, it is not clear how immune factors and persistent viral infection in the CNS lead to the differential outcomes of neurological sequelae. Moreover, small animal models to recapitulate TBEV-induced persistent neurological sequelae are under development. Due to the outbred nature and easy access, hamster models can serve as alternative small animal models to investigate neurotropic flavivirus infection. Neurological sequelae persist in a significant proportion of flaviviruses-infected convalescent patients. Continuous development of feasible animal models of neurological sequelae will facilitate the further investigation of the disease mechanisms. 

## Data Availability

Data sharing not applicable. No new data were created or analyzed in this study.
